# Controlled Transcription of Regulator Gene *carS* by Tet-on or by a Strong Promoter Confirms Its Role as a Repressor of Carotenoid Biosynthesis in *Fusarium fujikuroi*

**DOI:** 10.3390/microorganisms9010071

**Published:** 2020-12-29

**Authors:** Julia Marente, Javier Avalos, M. Carmen Limón

**Affiliations:** Department of Genetics, Faculty of Biology, University of Seville, 41012 Seville, Spain; jmarente@us.es (J.M.); avalos@us.es (J.A.)

**Keywords:** Tet-on, carotenoids, *Fusarium fujikuroi*, *carS*, *mluc* reporter gene, luciferase

## Abstract

Carotenoid biosynthesis is a frequent trait in fungi. In the ascomycete *Fusarium fujikuroi*, the synthesis of the carboxylic xanthophyll neurosporaxanthin (NX) is stimulated by light. However, the mutants of the *carS* gene, encoding a protein of the RING finger family, accumulate large NX amounts regardless of illumination, indicating the role of CarS as a negative regulator. To confirm CarS function, we used the Tet-on system to control *carS* expression in this fungus. The system was first set up with a reporter *mluc* gene, which showed a positive correlation between the inducer doxycycline and luminescence. Once the system was improved, the *carS* gene was expressed using Tet-on in the wild strain and in a *carS* mutant. In both cases, increased *carS* transcription provoked a downregulation of the structural genes of the pathway and albino phenotypes even under light. Similarly, when the *carS* gene was constitutively overexpressed under the control of a *gpdA* promoter, total downregulation of the NX pathway was observed. The results confirmed the role of CarS as a repressor of carotenogenesis in *F. fujikuroi* and revealed that its expression must be regulated in the wild strain to allow appropriate NX biosynthesis in response to illumination.

## 1. Introduction

Carotenoids are terpenoid pigments with essential roles in photosynthesis in autotrophic species, but they are also produced by some heterotrophic ones [1]. The latter include many fungi, which can produce and accumulate high levels of carotenoids [2]. This ability is of biotechnological importance, because some carotenoids perform critical functions in animals and humans, being the source of different biological molecules and having beneficial antioxidative properties [3,4]. Because animals and human are not able to synthetize carotenoids, they must obtain them in the diet [5].

The filamentous fungus *Fusarium fujikuroi*, a reference model for the research of fungal carotenogenesis, produces mainly a carboxylic xanthophyll called neurosporaxanthin (NX) [6]. The biosynthesis pathway of carotenoids in this fungus is well known, and all of the structural genes involved, called *car* genes, have been previously described. NX biosynthesis in *Fusarium* is induced by light and by nitrogen starvation, and both inducing effects are additive. The stimulation by light, achieved through the transcriptional induction of the structural *car* genes, has been investigated in detail [7]. In turn, the pathway is downregulated by the protein CarS, belonging to the RING finger family, and identified through the genetic characterization of *carS* mutants [8]. These mutants exhibit deep orange pigmentation under all culture conditions and accumulate large amounts of NX due to a strong upregulation of the structural *car* genes regardless of light [9,10]. Regulation by CarS especially affects the *car* cluster, consisting of the genes *carO*, *carB*, *carRA*, and *carX*. While *carB* and *carRA* encode two key enzymes of carotenoid biosynthesis, phytoene desaturase and phytoene synthase/carotene cyclase, *carO* and *carX*, encode a rhodopsin and a β-carotene cleaving enzyme, producing the CarO cofactor, retinal. The molecular mechanism of action of CarS is still not understood, but its similarity with other proteins with RING finger domains suggests a capacity to interact with E3 ligase-type enzymes that mediate ubiquitylation of target proteins. In fact, a RING finger protein, CrgA, represses carotenogenesis in *Mucor circinelloides* and its lack of function causes an over-accumulation of carotenoids [11]. Four orthologous CrgA genes were found in *Phycomyces blakesleeanus*, but only one of them could complement the *crgA* mutant of *M. circinelloides* [12].

The current hypothesis regarding the activity of CarS is focused on its possible interaction with other regulatory proteins to modulate their activity. Regulatory scenarios involving protein–protein interactions are not uncommon in the control of carotenogenesis in other microorganisms, even taxonomically distant, as exemplified by the complex regulatory network for the induction of carotenoid biosynthesis by light in myxobacteria [13]. In *Myxobacterium xanthus*, CarA and CarH are repressors that bind to the operator of a carotenoid operon in the dark. A third regulatory protein, CarS, acts as an antirepressor in the light, binds to CarA and CarH and disassembles them. The *F. fujikuroi* CarS protein has no structural relationship with the *M. xanthus* homonym, but it probably coincides in its ability to specifically interact with transcription factors involved in the control of structural *car* genes. CarH is homologous of LitR, a repressor widely distributed in nonphototrophic bacteria. Proteins of the CarH/LitR family play an important role as negative regulators of light-inducible carotenoid transcription genes and at the same time serve as photosensors [14]. Another repressor found in *Corynebacterium glutamicum* and *Actinobacteria* is CrtR, whose mutation causes the constitutive production of carotenoids independently of light [15,16]. CrtR represses its own gene and the *crt* operon by binding to the promoter sequence [15,16]. The activity of this repressor is modulated by geranylgeranyl pyrophosphate (GGPP), which means that it is capable of sensing the amount of this metabolite [17].

**Figure 1:**
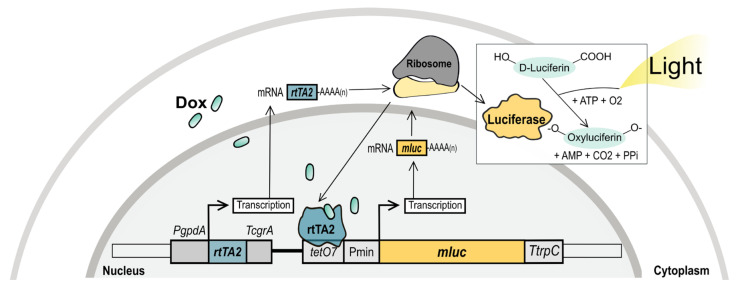
Model of the Tet-on mechanism to control the expression of *mluc*. Plasmid pVG3.1 contains the gene for the tetracycline-dependent transactivator *rtTA2^S^-M2* under control of the constitutive promoter of the glyceraldehyde-3-phosphate dehydrogenase gene from *Aspergillus nidulans* (P*gpdA).* In the presence of Dox, rtTA2^S^-M2 binds to the operator sequence *tetO7*, activates the fungal minimal promoter of P*gpdA* (Pmin), and consequently induces *mluc* gene expression. This gene encodes the enzyme luciferase that converts luciferin into oxyluciferin and emits light.

A recent RNAseq study revealed that *carS* mRNA levels are low under standard laboratory conditions, especially in the dark [18]. The objective of this work is to throw more light on CarS function through the overexpression of the *carS* gene using two strategies: (i) *carS* control by an inducible Tet-on expression system, and (ii) *carS* overexpression by the strong P*gpdA* promoter. Tet-on is an established bacterial regulatory system that was adapted to filamentous fungi to manipulate gene expression in *Aspergillus fumigatus* [19], and subsequently developed and improved in *Aspergillus niger* [20,21]. Its mechanism is displayed in Figure 1.

Briefly, the *A. nidulans* P*gpdA* promoter supports the constitutive expression of the tetracycline-dependent transactivator rtTA2^S^-M2. When rtTA2^S^-M2 is attached to the inducer doxycycline (Dox), it is able to bind to the operator sequence *tetO7* and activate the fungal promoter Pmin (a short version of P*gpdA*) and initiate the expression of the reporter gene *mluc* that encodes for the enzyme luciferase. In the presence of its substrate, luciferin, the luciferase emits light and produces oxyluciferin. This system has become a useful tool to control gene expression in many fungi [22,23], and was successfully used in *F. fujikuroi* to activate the silent trichosetin gene cluster [24]. Here, we used the Tet-on system to generate *F. fujikuroi* strains with tunable *carS* gene expression, which allowed to confirm the repressive role of this protein in carotenoid biosynthesis. The enhanced expression of *carS* through Tet-on or through the constitutive P*gpdA* promoter from *A. nidulans* resulted in an albino phenotype, with very low carotenoid production under illumination, indicating that *carS* expression is adapted to low levels for appropriate regulation of carotenoid biosynthesis in *Fusarium*.

## 2. Materials and Methods

### 2.1. Strains and Culture Conditions

The wild strain of *Fusarium fujikuroi* IMI58289 was obtained from the Imperial Mycological Institute (Kew, Surrey, England), and carotenoid overproducer mutant SG39 was isolated from IMI58289 by chemical mutagenesis [8]. Both strains and transformants from this work are listed in Table 1.

The strains were grown in DG medium, composed of 30 g glucose, 3 g NaNO_3_, 1 g KH2PO4, 0.5 g KCl, 0.5 g MgSO_4_·7H_2_O and 2 mL of microelements [25] per liter. Microelement are composed of 0.1 g FeSO_4_·7H_2_O, 0.015 g CuSO_4_·5H_2_O, 0.161 g ZnSO_4_·7H_2_O, 0.01-g MnSO_4_·7H_2_O, 0.01 g (NH_4_)6Mo_7_O_20_·4H2O in 100 mL of distilled water.

Strains were cultured at 30 °C for phenotypic and molecular analysis. For sporulation, they were grown in EG agar medium in Petri dishes and incubated under white light for 7 days at 26 °C. Composition of EG medium is 1 g glucose, 1 g yeast extract, 1 g NO_3_NH_4_, 1 g KH_2_PO_4_, 0.5-g MgSO_4_·7H_2_O and 16 g agar per liter [18]. Spores were harvested with water, separated from mycelia by filtration, and counted in a hemocytometer (Bürker chamber, Blau Brand, Germany). For luminescence assay, the strains were grown in DGpep, consisting of DG medium with 2 g/L of peptone.

For expression analysis, 100 mL of DG medium were inoculated with 10^6^ fresh spores of the corresponding strain in 500-mL flaks and incubated for 3 days in an orbital shaker at 150 rpm in dark. After this time, liquid cultures were distributed in four Petri dishes, and they were exposed to white light or incubated in the dark for 1 h, with a previous adaptation to the Petri dishes for 4 h in darkness. Illumination was performed under a platform with 4 fluorescent tubes (Philips TL-D 18 W/840) at ca. 60 cm, providing a light intensity of 7 W/m^2^ (420 Lm/w). For DNA isolation, cultures were incubated in a similar way, without specific illumination conditions. The mycelia were collected by filtration, frozen in liquid nitrogen, and stored at −80 °C until use.

For carotenoid determinations, the strains were grown at 30 °C on 25 mL of DG agar medium in standard Petri dishes for 7 days under illumination or in darkness. Strains were inoculated with sterile toothpicks at 7 symmetrical points on each Petri dish.

### 2.2. DNA Isolation and PCR Assays

Genomic DNA extractions were performed using the GenElute Plant Genomic DNA Miniprep kit (Sigma-Aldrich, St. Louis, MO, USA) following the manufacturer’s instructions. DNA quality was checked by gel electrophoresis and quantified in a Nanodrop ND-1000 spectrophotometer (Nanodrop Technologies, Wilmington, DE, USA). Two different DNA polymerases were used in PCR reactions. High fidelity DNA polymerase velocity (Bioline, Memphis, TN, USA) was used for plasmid construction and sequencing, while DNA polymerase BIOTAQ™ (Bioline GmbH, Germany) was used to check constructs and transformant candidates.

### 2.3. Generation of TETluc and TETcarS Transformants

To generate TET*luc* transformants, wild protoplasts were transformed with plasmids pVG3 (P*gpdA*::rtTA::T*cgrA*–*tetO7*::Pmin::*mluc*::T*trpC*) [20] digested with *Kpn*I, and PAN7-1, which contains the hygromycin resistance cassette, with *hph* gene, as a selection marker. After single-spore purification, we obtained three transformants which were analyzed by PCR, to check pVG3 integration, using two different combinations of internal primer pairs (Table S1). The three transformants gave a 1-kb band with primers Pmin-1F and mluc-1R and a band of 2.3 kb with rtTA2-1F and mluc-1R primers, indicating the integration of the Tet-on cassette (Figure S1).

To generate TET*carS* transformants, protoplasts from wild-type and SG39 mycelia were transformed with the linear pPO5 and the PAN7-1 plasmids, with the hygromycin resistance cassette [26], as described [27]. After the purification procedure in hygromycin-supplemented medium, two different kinds of transformants were obtained, seven originating from the wild strain and nine from the *carS* mutant strain. Both kinds of transformants were checked by PCR using the same pair of primers, Pmin-1F and CarS-9R, which should amplify a 1.6-kb band (Table S1, Figures S2 and S3). Three transformants from the *carS* mutant (T6, T7, T9) and four from the wild strain (T1, T5, T6, T7) showed the correct band and were considered as positive transformants.

### 2.4. Construction of pJM2 Plasmid and Generation of carS Constitutive Overexpression Transformants

For *carS* overexpression, the *carS* coding sequence was placed under the control of *gpdA* promoter (P*gpdA*). The P*gpdA::carS* fragment was generated by fusion PCR. P*gpdA* was amplified from PAN7-1 vector using primers PgpdA-Not-1F and PgpdA-carS-1R. The forward primer contained a *Not*I restriction site and reverse primer PgpdA-carS-1R included an overlapping sequence of 21 pb with the start codon of *carS* (Table S1). A 2.2-kb DNA fragment, containing the *carS* gene and part of the *trpC* terminator, was amplified from plasmid pPO5 using primers carS-PmeI-1F and trpC-MluI-2R with restriction sites for *Pme*I and *Mlu*I, respectively. Both fragments were fused with primers PgpdA-Not-1F and trpC-MluI-2R. The fusion product was purified and ligated to pGEM^®^-T easy vector (Promega), resulting in pJM1 plasmid. For pJM2 construction, plasmids pJM1 and PBN008 (containing the *amdS* cassette allowing the use of acetamide as nitrogen source) were digested with *Not*I and *Mlu*I restriction enzymes, and the purified DNA fragment (P*gpdA::carS*) was ligated to the digested PBN008 plasmid.

Wild protoplasts were transformed with the pJM2 plasmid following our standard protocol [27], and three transformants were isolated after the single-spore purification procedure in the selective medium with acetamide as nitrogen source. Transformants were analyzed by PCR, using primers S1carS-1F and amiE-1R that should amplify a 1.7-kb band that is found in all the candidates (Figure S4).

### 2.5. Southern Blot Analyses

Southern blot analyses were conducted using digoxigenin-labelled probes, which were amplified by PCR from genomic DNA and labelled with the DIG DNA Labelling Mix (Roche, Mannheim, Germany), following the manufacturer’s instructions. The sensitivity of the probes was checked before their use. Approximately 15 μg of genomic DNA from transformants were digested with different enzymes, electrophoresed in 0.7% agarose gels, and transferred by capillarity to a positively charged nylon membrane (Hybond-N from Amersham) as described [28]. The membrane was incubated with 25 mL of prehybridization solution: 5x Saline Sodium Citrate (5x SSC, containing 750 mM NaCl in 75-mM sodium citrate, pH 7.0), 0.7% sodium dodecyl sulfate (SDS), 0.1% N-lauroylsarcosine, and 2x Roche blocking reagent in maleic buffer (118.3 mM maleic acid, 150 mM NaCl, pH 7.5) at 50 °C in a glass cylinder for 1 h in a hybridization oven (HB-100 Hybridizer, UVP). After this, it was incubated overnight with the same solution containing 25 ng/mL of probe.

Afterwards, the membrane was washed twice with 2x washing solution: 2x SSC and 0.1% SDS for 5 min at 25 °C, and twice with 0.1x washing solution: 0.1x SSC and 0.1% SDS at 68 °C. After equilibrating the nylon with maleic buffer, it was blocked for 1 h. Then, 3-μL of antibody antidigoxigenin (Anti-Digoxigenin-AP, Fab fragments, Roche, Mannheim, Germany) was added to 30 mL of blocking solution and incubated for 30 min. The membrane was then washed twice with maleic buffer with 0.3% Tween-20 for 15 min and incubated between acetates for 2 min at room temperature in detection buffer. Detection was performed with CDP-Star^®^, ready-to-use (Roche), and signals were detected in an Odyssey Fc Imaging System (LI-COR, Lincoln, NE, USA).

### 2.6. Luminescence Assay

Luminescence emission from TET*luc* transformants (SG253 and SG255) was measured in Costar 96-well white clear-bottom plates (Corning, Corning, NY, USA) using the wild strain as a negative control. In each well, 50 μL of 2x DGpep was inoculated with 10^4^ fresh spores and incubated at 30 °C for 16 h. Afterwards, 50 μL of doxycycline, hereafter Dox, was added at different concentrations (2.5, 5, 10 and 20 μg/mL in water) to activate rtTA2^S^-M2.

A total of 50 μL of 0.2 mM luciferin (Promega, Madison, WI, USA), previously diluted in 2x DGpep medium, was added to each well with a final volume of 200 μL. For luminescence detection, measurements were periodically taken in a Multimodal Synergy HT plate lector for 55 h, and the absorbance at 600 nm was simultaneously measured. Data were analyzed in reference to absorbance.

### 2.7. Expression Analyses

All the strains were cultured in the dark except for the OE*carS* transformants, which were also exposed to light. Total RNA was isolated using the RNeasy Plant Mini Kit (Qiagen, Chatsworth, CA, USA). RNA samples were checked by electrophoresis and quantified by Nanodrop ND-1000 spectrophotometer (Nanodrop Technologies, Wilmington, DE, USA). Retrotranscription to cDNA was made with 2.5 μg of RNA, using the Transcriptor first-strand cDNA synthesis kit (Roche, Mannheim, Germany), and final cDNA concentrations were adjusted to 25 ng/μL.

The RT-PCR measurements were performed in a LightCycler 480 real-time instrument (Roche Mannheim, Germany) with the LightCycler 480 SYBR green I Master kit (Roche, Mannheim, Germany), following the manufacturer’s protocols. The primers used for the amplification and detection of the mRNA of genes *carS*, *carB*, *carRA*, *mluc*, FFUJ_04397 and *gpdA* are listed in Table S2. Expression values were normalized against those of the reference β_1_ tubulin and *gpdA* genes.

### 2.8. Carotenoid Measurements

For quantification of carotenoids, the strains were grown on DG agar medium, and in the case of the Tet-on transformants, the media also contained 20 µg/mL Dox, except for the control plates. After seven days of incubation at 30 °C under illumination, mycelia were removed from agar media with a clean scalpel blade and stored at 20 °C.

Carotenoids were extracted with acetone from lyophilized mycelia following a standard protocol [29], with two pulses of 6 m/s for 30 s in a FAST- PREP24 (Biomedicals, Irving, CA, USA). Extracted carotenoids were concentrated and measured as described [27].

### 2.9. Statistical Analysis

Unpaired and paired *t* Student tests were used to analyze differences in carotenoid accumulation and gene expression with GraphPad Prism 8 application (https://graphpad.com).

Values of *p* lower than 0.05 were considered significantly different. Levels of significance are indicated with asterisks: * indicates *p* < 0.05; ** indicates *p* < 0.01; *** *p* < 0.001 and **** *p* < 0.0001.

## 3. Results

### 3.1. Improvement of the Gene Expression Control by the Tet-on System in F. fujikuroi using a Reporter Gene

To test the Tet-on system as a tool to control transcription activation in *F. fujikuroi* and settle suitable experimental conditions for modulated *carS* expression, the wild strain was transformed with plasmid pVG3, previously used to establish the Tet-on system in *A. niger* [20]. The pVG3 plasmid contains the *mluc* gene, coding for the luciferase enzyme under the control of a minimal promoter regulated by the Tet-on system. Three transformants, SG253, SG254, and SG255, were generated, and the random insertion of plasmid pVG3 was confirmed by PCR and Southern blot hybridization (Figure S1).

**Figure 2:**
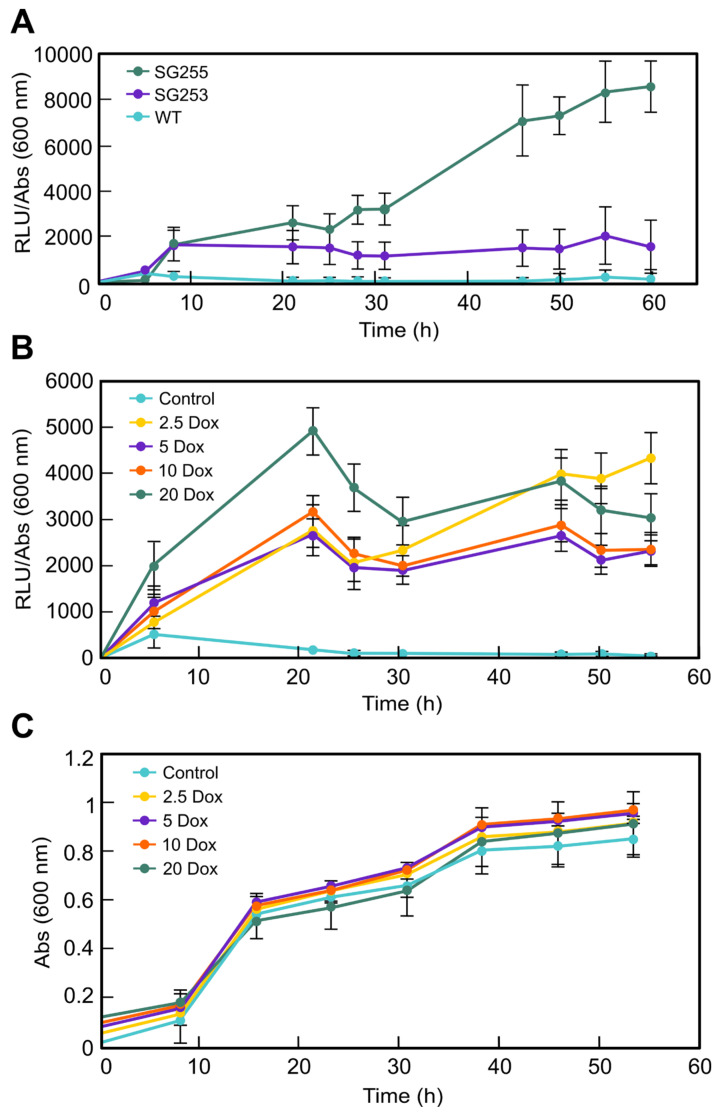
Luminescence emission of TET*luc* transformants induced with doxycycline (Dox) and compared to the wild strain: (**A**) The TETluc transformants (SG253 and SG255) and the wild strain (WT) were grown in DGpep medium for 16 h in 96-well plates; then, 0.2 mM luciferin and 20 μg/mL of Dox were added to the wells and incubated in the dark at 30 °C for the time indicated. Data are average and standard deviation for two independent experiments. As a negative control, strains were grown without Dox. (**B**) Luminescence emitted by the TET*luc* transformant SG255 in DGpep medium in 96-well plates. Dox was added at desired final concentrations (0, 2.5, 5, 10, and 20 μg/mL) to mycelia previously grown for 18 h. (**C**) Growth of SG255 in the 96-well plates described in panel B, measured by their absorbance at 600 nm. Data are average and standard deviations for three independent experiments.

Luminescence emission was only detected in SG253 and SG255, hereafter the TET*luc* transformants. The first assay, performed with 20 μg/mL Dox, showed different emission patterns for the two TET*luc* transformants (Figure 2A), suggesting differences in the integration events or in the genomic locations of the integrated plasmid. Because of its higher efficiency, TET*luc* transformant SG255 was selected to measure luminescence with different Dox concentrations (0, 2.5, 5, 10, and 20 μg/mL), and to study the activation of the Tet-on system.

The luminescence data obtained with each Dox concentration showed visible differences, but a similar trend was maintained over time with all the tested concentrations (Figure 2B). In this case, luminescence was particularly high at about 21 to 24 h with 20-μg/mL, but no important differences were observed with longer incubations compared to other Dox concentrations. The differences in luminescence generation by SG255 according to the Dox concentration were not explained by changes in growth, since only minor differences in mycelial density were observed in the wells with increasing Dox amounts (Figure 2C). The changes observed in cell density were not statistically different, as indicated by ANOVA test (Table S5). The optimal luminescence conditions found in these experiments were chosen to carry out experiments to modulate *carS* expression under control of the Tet-on system.

### 3.2. Use of the Tet-on System to Control carS Expression

**Figure 3:**
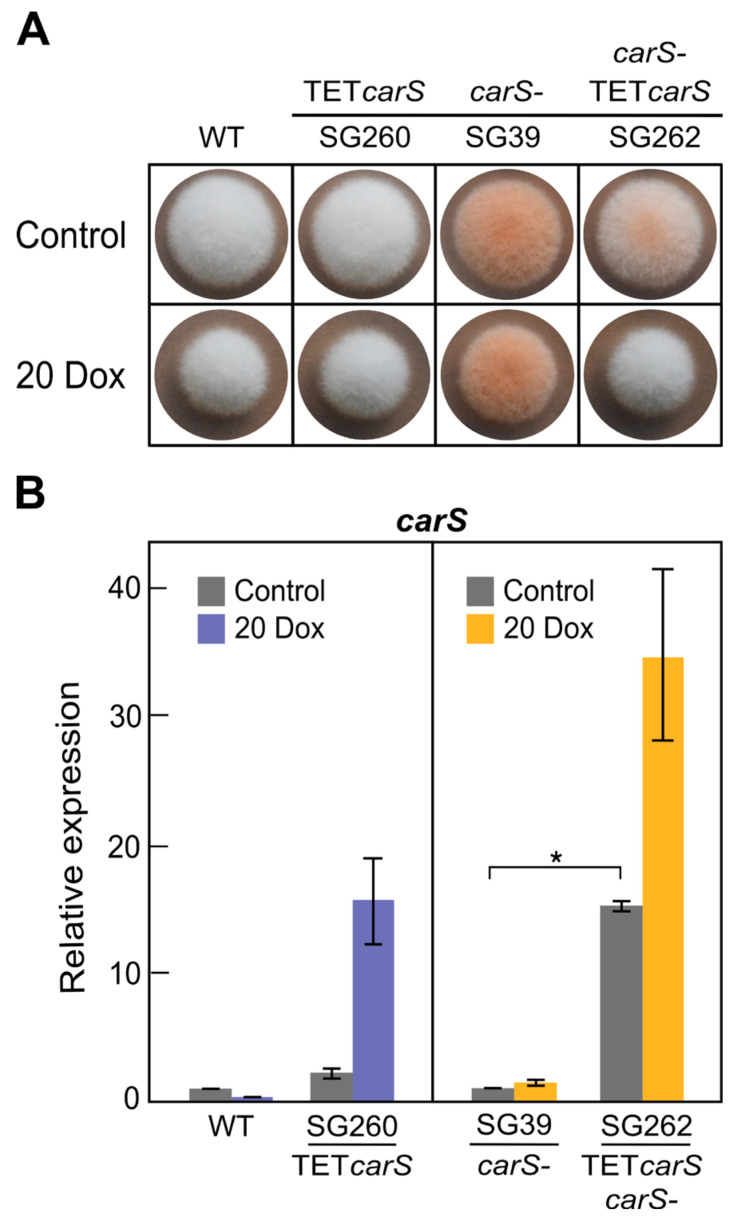
Control of expression of the *carS* gene using the Tet-on system in *F. fujikuroi*: (**A**) Phenotypic characterization of TET*carS* transformant (SG260 and SG262) and parental strains, wild type (WT) and SG39 (*carS* mutant), grown in DG agar medium (control) and in DG with 20 μg/mL Dox (20 Dox) at 30 °C for five days in the dark. (**B**) Transcripts levels of the *carS* gene in the strains grown in minimal DG medium for 18 h and induced with 20 μg/mL of Dox (20 Dox) or grown without Dox (control) and then incubated for up to three days in darkness. Values in the left graph refer to those of WT without Dox, and values in the right graph refer to those of SG39 without Dox. The qRT-PCR data are the average of two independent experiments and bars represent the standard deviation. *-indicates *p* < 0.05.

As stated above, mutations of the *carS* gene provoke an overproduction of carotenoids in *F. fujikuroi*, indicating a role of CarS as a negative regulator of carotenogenesis in this fungus. To deepen in the knowledge on this role, *carS* expression was controlled using the Tet-on system. The wild strain and *carS* mutant SG39 were transformed with plasmid pPO5, derived from pVG3 [27]. Four transformants obtained from the wild strain were confirmed to contain the construct by PCR, but only two, T1 and T5, showed the correct band pattern in Southern blot analysis (Figure S2). When these two strains were grown on DG agar medium with 20 μg/mL Dox, only T1, hereafter SG260, exhibited an albino phenotype in the presence of Dox in the light. On the other hand, three out of nine transformants derived from the *carS* mutant SG39 were found to contain the construct by PCR (Figure S3). As observed with SG260, the SG39-derived transformant T6, named SG262, showed an albino phenotype on DG agar with Dox addition (Figure 3A).

The effect of Dox addition on transcript levels of the *carS* gene was analyzed by qRT-PCR in SG260 and SG262 (Figure 3B). As a result, higher levels of *carS* mRNAs were observed when cultured with Dox compared to their corresponding parental strains (wild strain and SG39, respectively). The *carS* mRNA increased in SG260 in the absence of Dox, probably due to the basal expression of the Tet-on construct, which is added to that of the native *carS* gene. Unexpectedly, *carS* mRNA levels were particularly high in the SG39-derived transformant SG262 in the absence of Dox, and the strain had less pigmentation, suggesting that the increase in *carS* mRNA reduces the pigment synthesis. We conclude that the albino phenotype shown by SG262 in the presence of Dox is due to the enhanced levels of CarS protein under these conditions.

### 3.3. Effect of Control of carS Expression on Carotenogenesis in the carS Mutant

Because of its high carotenoid content and the absence of a wild-type *carS* allele, the effect of controlling *carS* expression on carotenogenesis is more amenable to follow in the *carS* mutant SG39. For this reason, we analyzed the carotenoid content and the expression of two structural genes of carotenogenesis in mycelia of the *carS* mutant SG39 and its transformant SG262 grown in darkness. Because of their strong regulation by CarS, the *carB* gene**, which encodes the phytoene desaturase, and the *carRA* gene, which encodes the phytoene synthase/carotene cyclase, were chosen to study the effect of the increase in *carS* mRNA.

**Figure 4:**
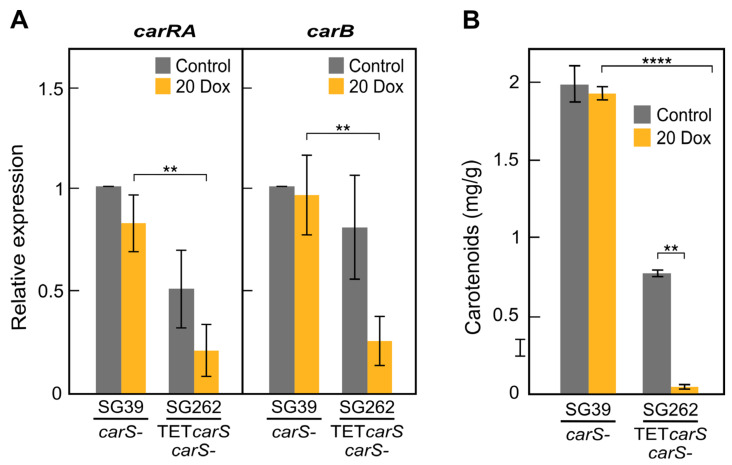
Repression of the carotenoid biosynthesis pathway by *carS* overexpression in the *carS* mutant: (**A**) Transcripts levels for the *carB* and *carRA* genes in the TET*carS* transformant (SG262) and the *carS* mutant (SG39). Cultures of SG39 and SG262 grown for 18 h were induced with 20 μg/mL of Dox and then incubated for up to three days in darkness. The noninduced controls were grown for three days without Dox. Quantitative RT-PCR values represent average and standard deviations of two independent experiments. (**B**) Carotenoid content from the same strains grown on DG agar medium with 20 μg/mL of Dox, and without Dox as control, for seven days in darkness. Mycelial samples were taken from three independent experiments. ** *p* < 0.01; **** *p* < 0.0001.

As expected, high *carB* and *carRA* transcript levels were found in the *carS* mutant SG39 regardless of Dox addition (Figure 4A). Induction of *carS* mRNA by Dox in the TET*carS* transformant SG262 provoked a decrease in the expression of *carB* and *carRA* (Figure 4A). Accordingly, the transformant SG262 did not produce carotenoids due to the strong activation of *carS* expression in the dark (Figure 4B). The carotenoid content was, however, notably reduced in the transformant in the absence of Dox, probably due to basal *carS* expression from the Tet-on system (Figure 3B) and to a local effect of the genomic integration. Although the decrease in *carB* expression did not significantly decrease in SG262 in comparison to SG39 in control media, *carRA* transcripts were reduced to half. The CarRA enzyme contains the phytoene synthase domain required for the synthesis of colored carotenoids. A lower amount in this enzyme could also explain a reduction in the final NX product.

**Figure 5:**
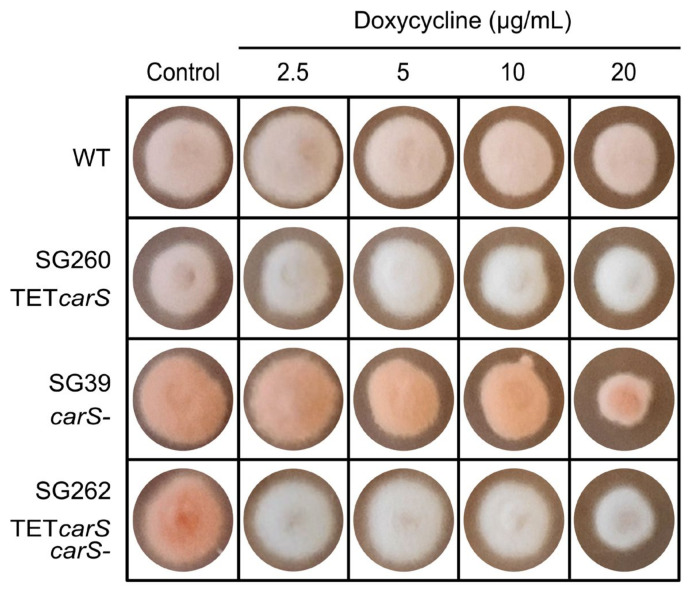
Growth and pigmentation of strains induced with a range of Dox concentrations. TET*carS* transformants (SG260 and SG262), the wild strain (WT) and *carS* mutant (SG39) were grown on minimal DG agar medium with different Dox concentrations (0, 2.5, 5, 10, and 20 μg/mL) at 30 °C, for five days under light.

In contrast to the wild strain and the transformants SG260 and SG262, the *carS* mutant SG39 exhibited slower growth in the presence of 20 μg/mL Dox in the light, indicating that this strain is more stressed than the others under this condition. To check the sensitivity of the *carS* mutant, we investigated the effect of different Dox concentrations on SG39 growth in comparison to the other strains (Figure 5). The results confirmed the reduced growth of SG39 with 20 μg/mL Dox, also detected to a minor extent for SG262 at the same concentration, but hardly exhibited by the wild strain and its derived SG260 TET*carS*-expressing transformant. However, no differences in growth were detected bet-ween the four strains at lower Dox concentrations.

**Figure 6:**
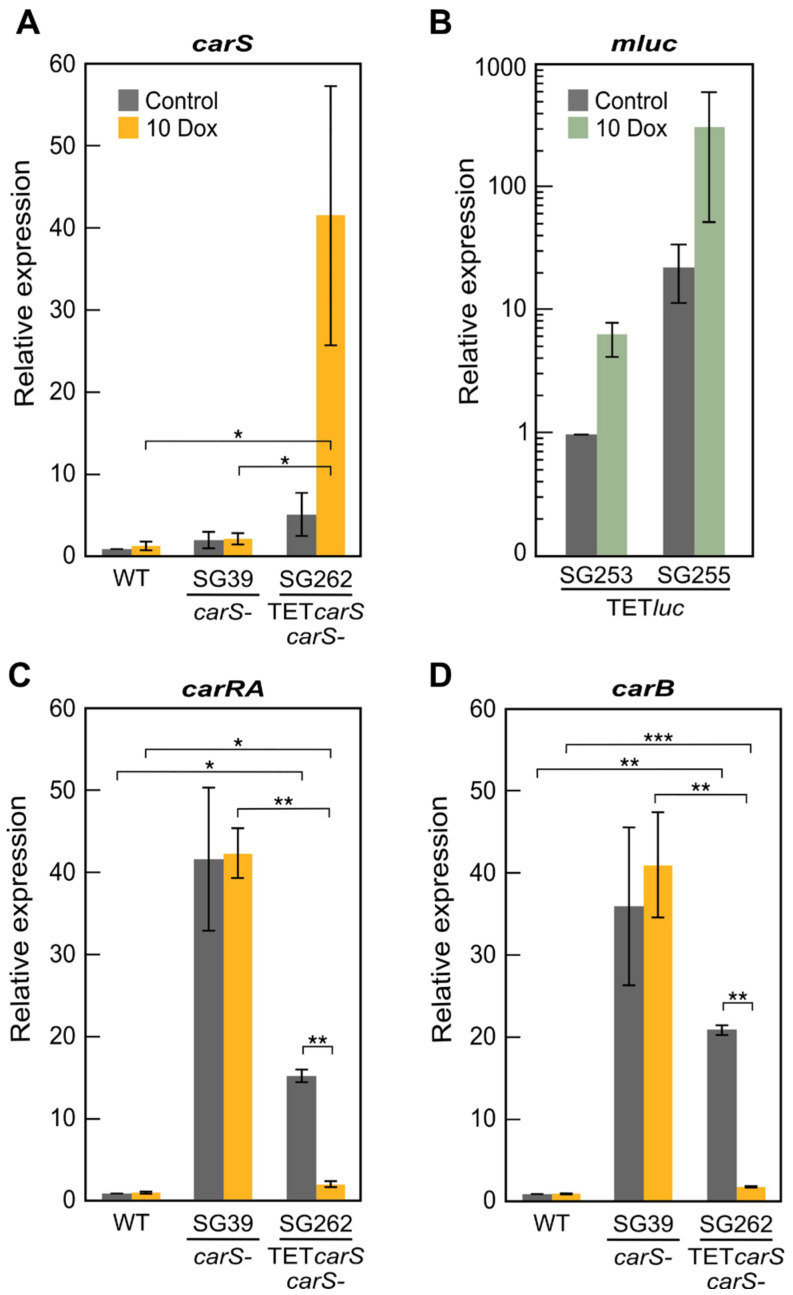
Effect of optimized Dox concentration on gene expression: (**A**) Transcripts levels for the *carS* gene in the wild strain (WT), *carS* mutant (SG39), and TET*carS* transformant (SG262). (**B**) Transcript levels for the *mluc* gene in TET*luc* transformants SG253 and SG255. Values were normalized to those of SG253 in the absence of Dox. (**C**) Transcript levels for the *carRA* gene in WT, SG39, and SG262. (**D**) Transcript levels for the *carB* gene in WT, SG39 and SG262. The strains were grown for two days in DG medium in darkness, then induced with 10 μg/mL Dox (10 Dox) for 24 h; noninduced cultures (control) were incubated for the same time. Data of qRT-PCR are the average and standard deviation of three independent experiments. Values were normalized to those of the noninduced wild strain, except for the *mlu*c gene. * *p* < 0.05; ** *p* < 0.01; *** *p* < 0.001.

The growth inhibition of SG39 and SG262 observed in Figure 5, in the presence of 20-μg/mL, suggests that these conditions are too stressful for these strains. For this reason, to use physiological conditions more similar to those of the control, new experiments were conducted, halving the amount of Dox and adding it to 48-h old cultures, which allowed induction to be carried out for 24 h. When the TET*carS* transformant SG262, the *carS* mutant SG39, and the wild strain were induced with 10 μg/mL Dox under these experimental conditions, there was no variation of *carS* transcript levels in the wild strain and SG39 in comparison to the control without Dox, while in SG262, there was an eight-fold increase (Figure 6A). However, the increase in *carS* mRNA in SG262 was only two-fold when induced with 20 μg/mL Dox (Figure 3B). As stated above, the structural genes *carRA* and *carB* were chosen as representatives of *carS*-regulated genes of carotenogenesis. Data were normalized to those of the wild strain in order to detect a presumptive downregulation caused by CarS. The results confirmed that the levels of mRNA of the *carRA* and *carB* genes in the *carS* mutant SG39 were not affected by 10 μg/mL Dox in the medium.

However, they showed a strong downregulation of their mRNA levels in the SG262 strain with this Dox concentration, approximately 7-fold for *carRA* and 12-fold for *carB* (Figure 6C and 6D), even more drastic that the 3-fold and 2-fold decrease found for the same genes with 20 μg/mL of the inducer in the same transformant (Figure 4A). Experiments were also conducted with the TET*luc* transformants SG253 and SG255 with 10 μg/mL of Dox in the dark to study *mluc* expression. As expected, no *mluc* mRNA was detected in the wild strain under the same conditions, and the transformants showed a correlation between their *mluc* transcript levels (Figure 6B) and their luminescence emission pattern (Figure 2A), which explains the higher levels of luminescence emission detected in SG255.

### 3.4. Effect of Constitutive carS Overexpression on the Carotenoid Biosynthesis Pathway

Due to the culture limitations and the possible influence of Dox on the strains as a stressing agent, we used an alternative strategy to investigate the effect of *carS* overexpression. Plasmid pJM2 was constructed, containing the coding *carS* sequence under control of the *A. nidulans* P*gpdA* promoter and the *amdS* gene as selection marker.

**Figure 7:**
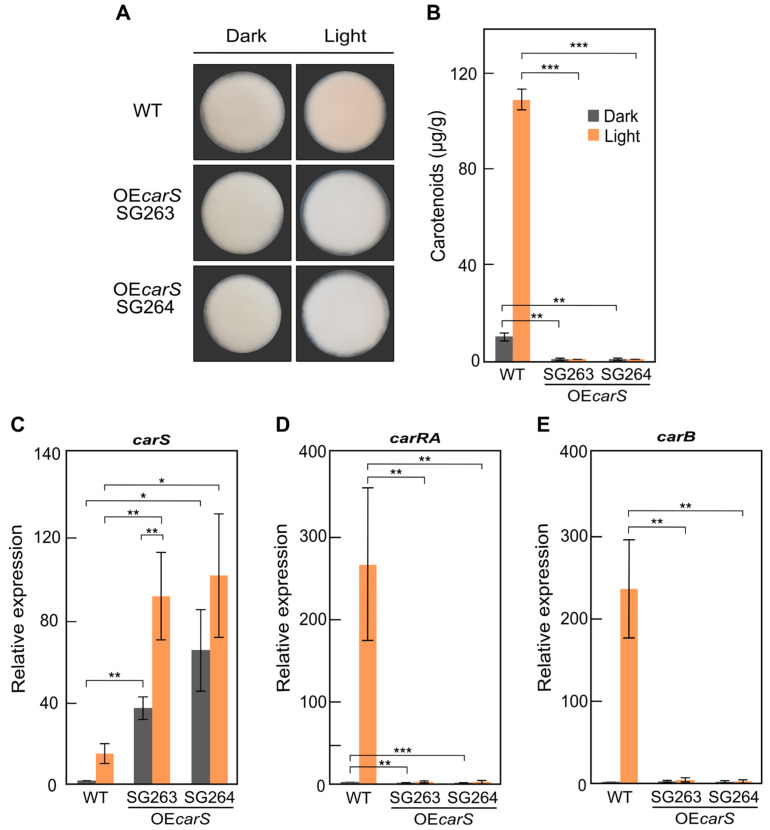
Effect of constitutive expression of the *carS* gene in *F. fujikuroi:* (**A**) Phenotypes of two OE*carS* transformants (SG263 and SG264) and the wild strain (WT) grown on DG agar medium for five days at 30 °C in the dark or under light. (**B**) Carotenoid content in mycelia of WT and OE*carS* transformants grown for seven days in darkness or under light. Data are the average and standard deviation from three independent experiments. (**C**–**E**) Transcript levels of *carS*, *carRA*, and *carB* in WT and OE*carS* transformants; RNA was isolated from strains grown in darkness for three days and exposed to light for one hour or kept for this time in the dark. Data of qRT-PCR are the average and mean error of five independent experiments. Values were normalized to the WT samples in the dark. * *p* < 0.05; ** *p* < 0.01; *** *p* < 0.001.

The wild strain was transformed with plasmid pJM2, and three transformants were analyzed. Two of them contained the P*gpdA-carS* construct, as confirmed by the results obtained from PCR amplification with appropriate primers and the correct bands observed in a Southern blot hybridization (Figure S4). Transformants SG263 and SG264 are albino either in darkness or under light (Figure 7A), and no carotenoids could be detected in their mycelia (Figure 7B). In accordance with this result, *carB* and *carRA* mRNA levels were practically indetectable in any of the transformants, and no induction by light could be observed (Figure 7D). The amount of *carS* mRNA detected by qRT-PCR was higher in SG263 and SG264 (Figure 7C) than in the two TET*carS* transformants upon Dox induction (Figure 6A). We conclude that *carS* overexpression results not only in the loss of the photoinduction of carotenoid production but also in a repression of the synthesis in the dark, indicating a role of CarS as repressor of the pathway irrespective of the regulation by light.

## 4. Discussion

The Tet-on system is a highly versatile tool to control gene expression that has been successfully used in different filamentous fungi, such as *A. niger*, *A. fumigatus*, *A. terreus* [21,22,30,31,32,33], and *F. fujikuroi* [24], among others. In the latter, a gene for transcription factor TF22 from a silent cluster was expressed at different levels using the bacterial–fungal hybrid promoter *tetO7*::P*poliC* system, and in TET::*TF22*, upregulation of three genes of the cluster for trichosetin was achieved [24].

In our case, the expression system was tuned up in TET*luc* transformants with the reporter gene *mluc* under the control of the *tetO7*::Pmin promoter. TET*luc* transformants have the advantage that luciferase activity is easy to measure, and they produced high luminescence in just few hours after the induction with Dox (Figure 2A). The rapid and strong induction of *mluc* in these strains is comparable to that formerly observed in similar transformants of *A. niger* [20]. The luciferase activity had a significant induction with 20 µg/mL Dox, and it could predictably keep increasing with higher concentrations. In fact, in other studies, high inductions were achieved with 50 µg/mL Dox; however, at that concentration, the growth of wild-type *F. fujikuroi* was appreciably inhibited on complete agar medium [24]. We detected that inhibition occurred with only 20 µg/mL Dox in solid cultures on minimal medium in the *carS* mutant SG39 and its derived transformant, but not in the wild type or its derived strain (Figure 3 and Figure 5). However, in microtiter plates, the inhibition with 20 µg/mL was not statistically significant, although the luciferase activity was outstanding. Moreover, optimization of culture conditions, such as aeration, culture composition, and illumination, reduces the toxicity caused by this tetracycline-derived antibiotic, which has been described as a selective inhibitor of mitochondrial protein translation [34]. For this reason, we recommend checking the effect of this antibiotic on the growth of the organism object of experimentation before using this Tet-on system.

Tet-on is an experimentally convenient inducible expression system that has been used for different purposes in different biological contexts. In has been used to express a polycistronic mRNA in *A. niger* [35] and to control gene expression by blue light- and Dox-dependent manner in mammalian cells [36]. One of the advantages of the Tet-on approach is that it allows studying phenotypes that could not be observed when the high expression of a regulator, an activator, or a repressor affects the viability of the transformant due to the production of secondary metabolites. We predict that this system will continually improve and will be used for new applications in the future to study gene regulation and to control the expression of genes for the production of metabolites of interest.

Since the identification of the *carS* gene, resulting from the analysis of mutations responsible of the deep orange pigmentation of a class of *F. fujikuroi* mutants [9], several studies have been performed to understand its role in the regulation of carotenoid biosynthesis [7]. This pathway is mainly regulated by light and to a minor extent by other environmental signals, such as nitrogen availability. The major regulatory protein involved in the regulation by light is WcoA, coding for a photoreceptor of the white-collar family [37]. However, the pathway is downregulated by the CarS protein, as indicates the strong increase in the mRNA levels of the *car* genes in the *carS* mutants [7]. Such mutants, however, still respond to light, and the possible involvement of CarS in the regulation mediated by WcoA remains to be investigated.

RNA-seq data on the effects of light and *carS* mutation in *F. fujikuroi* showed a high overlap between the genes regulated by light and those differentially expressed in the *carS* mutant, suggesting regulatory connections [18]. This indicates that CarS plays a role as a modulator of many light-regulated genes, but it does not imply a direct participation of CarS in the control by light. In this study, the increase in *carS* mRNA using either a Dox-inducible Tet-on promoter or a constitutive P*gpdA* promoter gave albino phenotypes. The results were similar with both strategies and showed that the increased levels of *carS* correlated with a complete reduction of mRNA levels of the structural genes *carRA* and *carB*. This is consistent with a dose-specific action of CarS, in which an excess of CarS protein results in an over-repressed carotenoid pathway. A partial repression is already operating in the wild strain, with presumable scarce CarS levels, as indicated by the low number of transcripts detected in the RNAseq studies and the high expression of the genes of the carotenoid pathway in the absence of the functional CarS protein. A novel lncRNA gene, located upstream to *carS*, may be at least partially responsible for the attenuated *carS* expression [38]. However, mRNA levels do not necessarily correlate with functional protein levels, since regulatory proteins are frequently the subject of interactions with other proteins that generate posttranslational modifications to modulate their activity or trigger their degradation. As an example, the white-collar protein WC-1, the major transcriptional activator of carotenogenesis in *Neurospora crassa*, is phosphorylated together with its WC-2 partner in the white-collar complex after activation by light, impairing its activating function [39]. The protein CarS is likely to be subject to a similar regulation. While its mRNA levels are higher after illumination, the carotenoid levels do also increase, which is the opposite effect that we would expect from a higher *carS* expression. In our case, however, the decreased carotenoid biosynthesis under CarS overproduction was also observed in the light.

In this study, the levels of *carS* in the OE*carS* strains were higher that with the Tet-on system and as a result provoked a complete repression of *carB* and carotenoid synthesis, while in the TET*carS* transformants, this gene was not fully repressed with the assayed Dox concentration. These results agree with other studies in which overexpression of a transcription factor with a constitutive promoter gave a higher production of trichosetin than with a Dox-inducible hybrid promoter [24]. Our findings reinforce the idea that the physiological levels of CarS are precisely tuned to maintain the amounts of carotenoids in the dark at low levels while still allowing a sufficient induction of the synthesis under illumination. Moreover, our data are consistent with the occurrence of a posttranscriptional regulation of *carS*, as indicated by the higher levels of *carS* mRNA in the light when the *carS* gene is overexpressed in the OE*carS* strains, considering that the P*gpd* promoter is not regulated by light. This increase could not to be explained by the *carS* expression from the native promoter, still present in the genome for the *carS* wild-type allele, because although relative *carS* mRNA values are higher in the wild strain in the light compared to the dark, the increase is quantitatively much higher in the SG263 and SG264 strains. As CarS is a repressor of *car* genes, the existence of a regulatory system to counteract the activating effect of light would be expectable, possibly involving a higher stability of *carS* transcripts in the light, but not necessarily implying a higher availability of the active CarS protein.

The fine regulation of carotenogenesis by the CarS protein in *F. fujikuroi* and its connections with the control by light is an intriguing scientific issue with potential biotechnological applications. Because of its antioxidant properties [4], NX is an attractive product for biotechnologists, and the understanding of the molecular mechanism that controls its synthesis in this fungus may have future applications. In this respect, the understanding of the posttranscriptional mechanisms governing *carS* function will be of particular interest in future research. For this, and for other purposes, the successful use of inducible expression systems, such as the Tet-on used in this work, is also very promising.

## Figures and Tables

**Table 1:** Strains used in this work.

Strain	Genotype	Code
IMI58289	Wild type	WT
SG39	*carS*-	*carS-*
SG253	*tetO7*::*Pmin*::*mluc hph+*	TET*luc*
SG255	*tetO7*::*Pmin*::*mluc hph+*	TET*luc*
SG260	*tetO7*::*Pmin*::*carS hph+*	TET*carS*
SG262	*tetO7*::*Pmin*::*carS carS- hph+*	TET*carS carS-*
SG263	P*gpdA*::*carS amdS+*	OE*carS*
SG264	P*gpdA*::*carS amdS+*	OE*carS*

## Supplementary Materials

The following are available online at https://www.mdpi.com/2076-2607/9/1/71/s1: Figure S1: Molecular analysis of transformants TET*luc* of *F. fujikuroi*; Figure S2: Molecular analysis of transformants TET*carS* of wild *F. fujikuroi*; Figure S3: Molecular analysis of transformants TET*carS* derived from the SG39 *carS* mutant; Figure S4: Molecular analysis of OE*carS* transformants of wild *F. fujikuroi*; Table S1: Primers used for the generation of the constructs, probes and verification of DNA integration in the transformants; Table S2: Sequences of primers used for quantitative RT-PCR, from 5′ to 3′; Tables S3–S18: Values of *p* for statistical significance after pairwise comparison of the data shown in the matrixes. The experiment from which the data were taken is indicated in each table.
