# Supplementary material for: Controlled Transcription of Regulator Gene carS by Tet-on or by a Strong Promoter Confirms Its Role as a Repressor of Carotenoid Biosynthesis in Fusarium fujikuroi
Source: Microorganisms. 2020 Dec 29;9(1):71. doi: 10.3390/microorganisms9010071 (PMC7824685; doi:10.3390/microorganisms9010071)
Supplement: Supplementary file 1 [file microorganisms-09-00071-s001.zip › Suppl final/Supplementary material_final.docx]

**Controlled Transcription of Regulator Gene *carS* by Tet-on or by a Strong Promoter Confirms Its Role as a Repressor of Carotenoid Biosynthesis in *Fusarium fujikuroi***

Julia Marente, Javier Avalos, and M. Carmen Limón

**SUPPLEMENTARY MATERIAL**

**Table S1.** Primers used for the generation of the constructs, probes and verification of DNA integration in the transformants.

| **Primer** | **Sequence 5' to 3'** | **Gene** |
| --- | --- | --- |
| Pmin-1F | GAGCAGACATCACCGTTTAAA | Pmin |
| rtTA-1F | CTGCGGAACAACGCCAAGTCA | *rtTA2* |
| mluc-1R | GATGGAACCTCTTGGCAACC | *mluc* |
| CarS-9R | GCAGGGGAGGCTGATGGGCT | *carS* |
| Foxy-2R | GACGATGTTAGTCTCGAGGA | *carS* |
| S1carS-1F | GCCAACTTTCATCAGCTAGG | *carS* |
| amiE-1R | ACGCTCCCTAGCAACACCTG | *amdS* |
| PgpdA-NotI-1F | AGGAAAAAAGCGGCCGCAAAAGGAAAAGGTGCAGTGGATGATTATTAATC | P*gpdA* |
| PgpdA-carS-1R | **ATTGCGTGCGTATAGTACGTTTAAAC**TGGGGTGATGTCTGCTCAAGCG | P*gpdA* |
| carS-PmeI-1F | CCAGTTTAAACGTACTATACGCACGCAAT | *carS* |
| TrpC-MluI-2R | AAAACGCGTTTTATTCTTGTTGACATGGAGCT | T*trpC* |

Restriction sites for *Not*I, *Pme*I and *Mlu*I are underlined

Overhanging nucleotides for fusion PCR are in bold

**Table S2.** Sequences of primers used for quantitative RT-PCR, from 5’ to 3’

| **Gene** | **Forward** | **Reverse** |
| --- | --- | --- |
| *mluc* | GCTCCAACACCCCAACATCTT | CGTCTTTCCGTGCTCCAAAA |
| *carS* | GATACCCGGCGGAAAGGTTA | CTGACAGTCCATTTCAGCGC |
| *carB* | TCGGTGTCGAGTACCGTCTCT | TGCCTTGCCGGTTGCTT |
| *carRA* | CAGAAGCTGTTCCCGAAGACA | TGCGATGCCCATTTCTTGA |
| *β_1_tub* | CCGGTGCTGGAAACAACTG | CGAGGACCTGGTCGACAAGT |
| *gpdA* | GTGACCTCAAGGGCGTTCT G | CGAAGATGGAGTTTGTGTT |

*β_1_tub* gene corresponds to FFUJ_04397


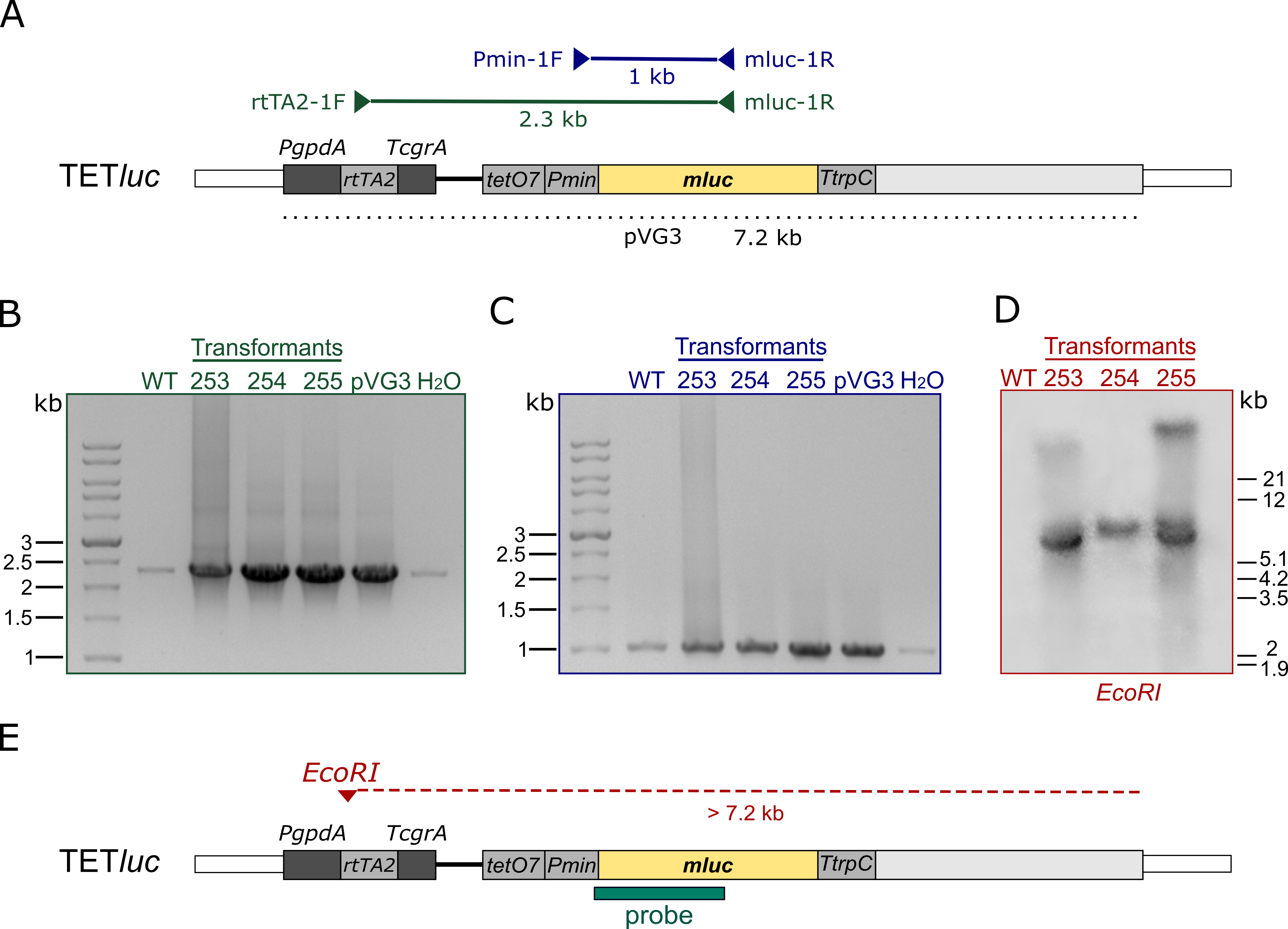


**Figure S1.** Molecular analysis of transformants TET*luc* of *F. fujikuroi:* (**A**) Map of genomic pVG3 integration region of TET*luc* transformants. Arrowheads indicate primers and solid lines correspond to PCR amplicons; (**B**) Electrophoresis of PCR products resulting from amplification with genomic DNA from wild strain (WT) and transformants TET*luc* (SG253, SG254, and SG255) using primers rtTA2-1F and mluc-1R. Plasmid pVG3 was used as a positive control; (**C**) Electrophoresis of PCR products resulting from amplification with genomic DNA from WT and transformants TET*luc* (SG253, SG254, and SG255) using primers Pmin-1F and mluc-1R; (**D**) Hybridization of Southern blot of WT and transformants SG253, SG254, and SG255. Genomic DNA was digested with *Eco*RI and hybridized with a 1-kb probe amplified with the primers Pmin-1F and mluc-1; (E) Restriction map of TET*luc* transformants with integrated plasmid pVG3. *Eco*RI site is indicated with a downward arrowhead. A fragment of *mluc* gene was used as a probe for the Southern hybridization.


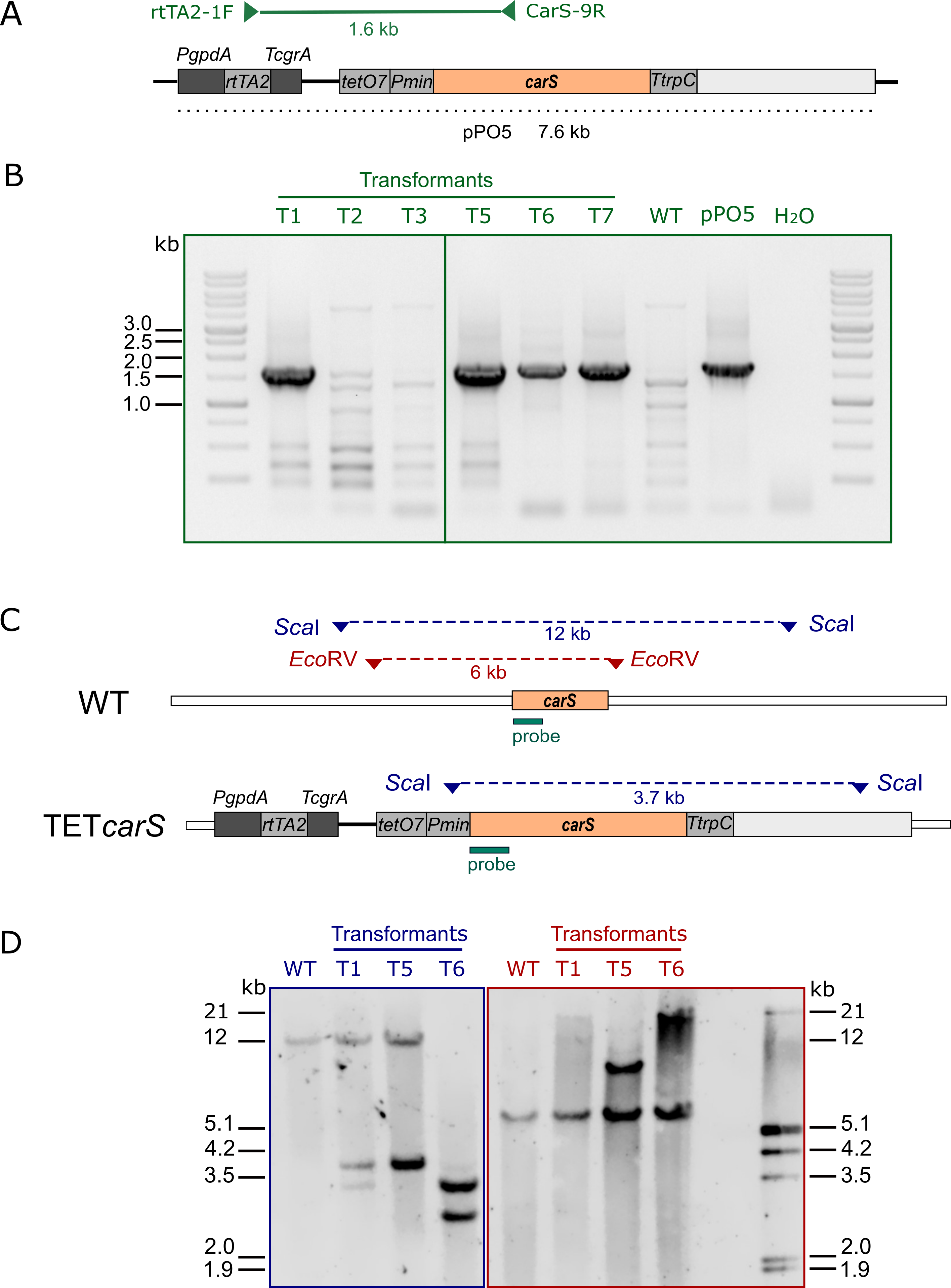


**Figure S2.** Molecular analysis of transformants TET*carS* of wild *F. fujikuroi:* (**A**) Map of genome with integrated pPO5 plasmid in TET*carS* candidate transformants. Green continuous line represents amplicon after PCR with primers rtTA-1F and CarS-9R. Primers are represented by arrowheads at the end of the lines; (**B**) Electrophoresis after PCR amplification using primers rtTA-1F and CarS-9R of genomic DNA from wild strain (WT) and transformant candidates: T1, T2, T3, T4, T5, T6, and T7. Those showing a 1.6-kb band were considered positives. Plasmid pPO5 was used as positive control. (**C**) Genomic region of WT and transformants TET*carS* with integration of plasmid pPO5. Enzyme restriction sites are indicated with triangles. Discontinuous lines represent DNA fragments after digestion with enzymes *Sca*I and *Eco*RV. Sizes are indicated below the lines. Code of colours: Red for *Eco*RV and blue for *Sca*I; (**D**) Southern blot of WT and transformants TET*carS* genomic DNA digested with *EcoR*V and *Sca*I, and hybridized with a 1.4-kb probe amplified with primers Pmin-1F and CarS-9R. Transformant T1 is SG260. T5 is SG261.


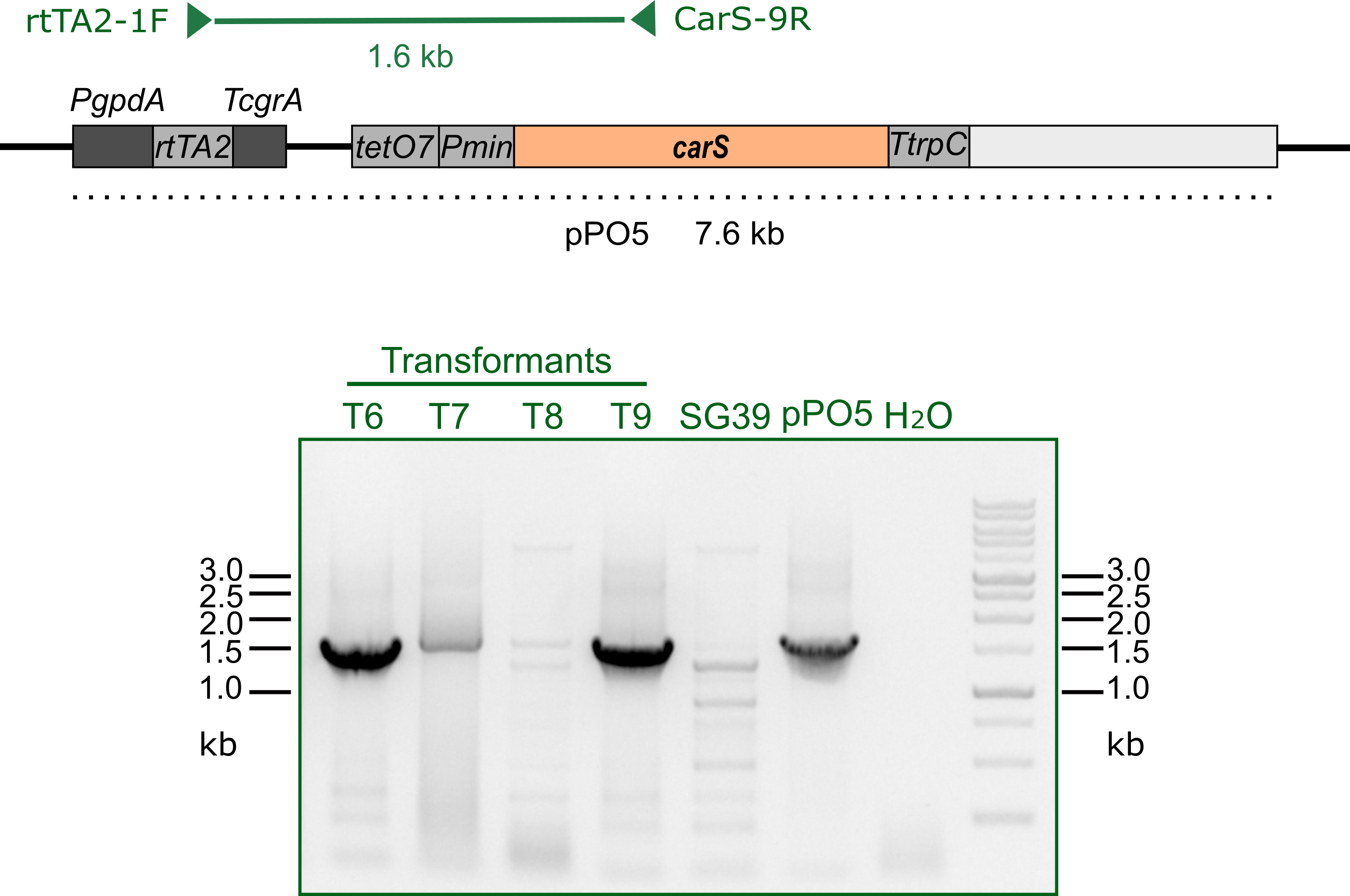


**Figure S3.** Molecular analysis of transformants TET*carS* derived from SG39 *carS* mutant: Map of genomic region with integrated pPO5 plasmid. Green continuous line represents the 1.6-kb amplicon expected in positive transformants after PCR amplification with primers rtTA-1F and CarS-9R. Green arrowheads at the end of the line represent the primers; PCR results of the amplification of genomic DNA from SG39 mutant and transformant candidates T6, T7, T8, and T9. DNA from pPO5 plasmid was used as positive control. T6 was named SG262 and chosen as representative TET*carS* transformant derived from SG39 for future analysis.


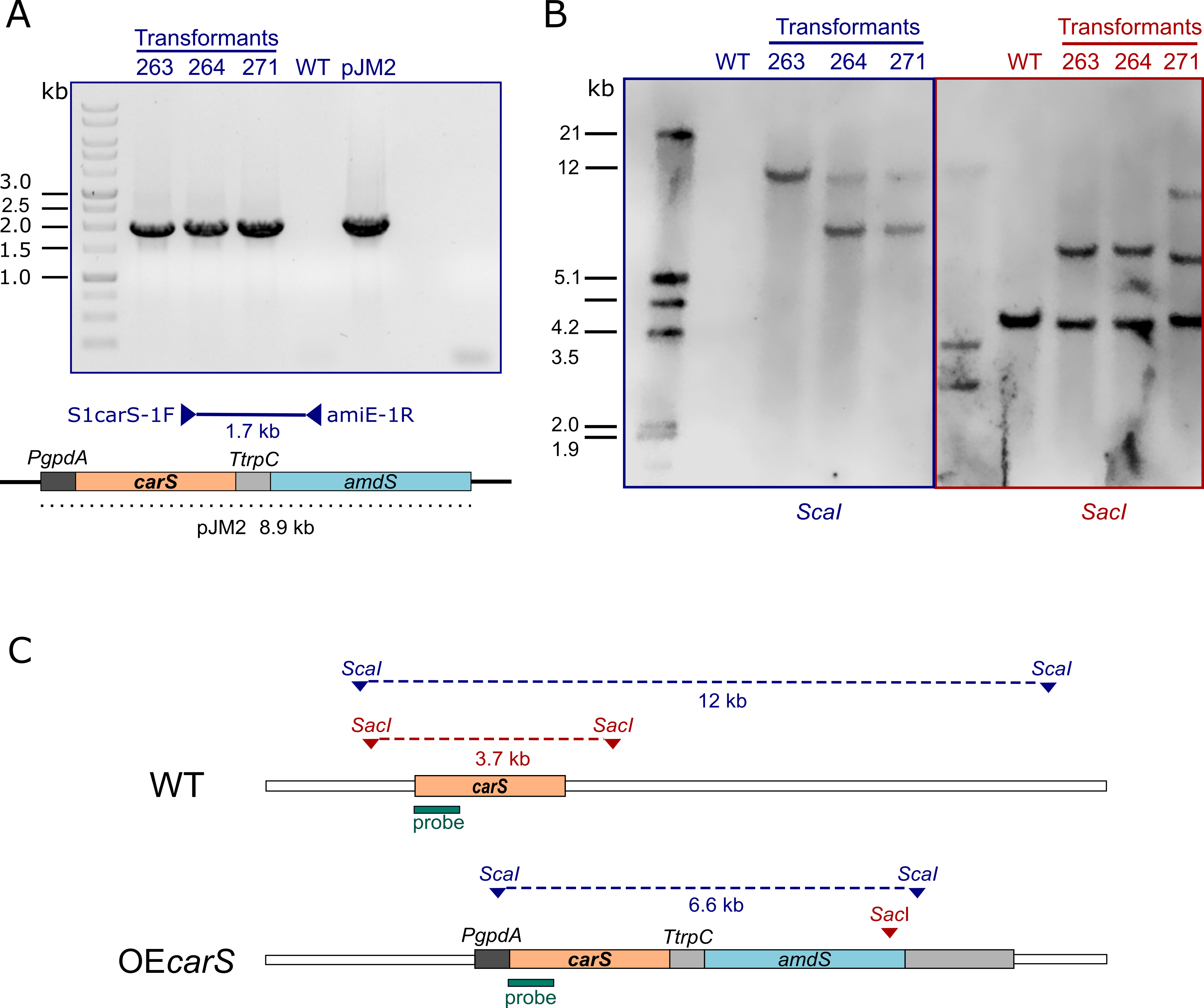


**Figure S4.** Molecular analysis of OE*carS* transformants of wild *F. fujikuroi:* **(A**) PCR amplification from wild strain (WT) and transformants SG263, SG264 and SG271 using primers S1carS-1F and amiE-1R of gDNA. Plasmid pJM2 was used as positive control. Below, map showing pJM2 plasmid integrated in OE*carS* transformants. Blue continuous line represents amplicon after PCR with primers S1carS-1F and amiE-1R. Primers are represented by arrowheads; (**B**) Southern blot of WT and OE*carS* transformants genomic DNA digested with *Sca*I or *Sac*I and hybridized with a probe for *carS* gene; (**C**) Genomic region of WT and transformants OE*carS* with integration of plasmid pJM2. Enzyme restriction sites are indicated with triangles. Discontinuous lines represent DNA fragments after digestion with enzymes *Sca*I and *Sac*I. Sizes are indicated below the lines. Code of colours: Blue for *Sca*I and red for *Sac*I. Transformants SG263 and SG264 were studied in more detail in this work.
