# Supplementary material for: Controlled Transcription of Regulator Gene carS by Tet-on or by a Strong Promoter Confirms Its Role as a Repressor of Carotenoid Biosynthesis in Fusarium fujikuroi
Source: Microorganisms. 2020 Dec 29;9(1):71. doi: 10.3390/microorganisms9010071 (PMC7824685; doi:10.3390/microorganisms9010071)
Supplement: Supplementary file 1 [file microorganisms-09-00071-s001.zip › Suppl final/Supplementary Tables S3-S17.docx]

**Controlled Transcription of Regulator Gene *carS* by Tet-on or by a Strong Promoter Confirms Its Role as a Repressor of Carotenoid Biosynthesis in *Fusarium fujikuroi***

Julia Marente, Javier Avalos, and M. Carmen Limón

**SUPPLEMENTARY MATERIAL**

**Table S3.** Statistical significance (p values) obtained after pairwise comparisons of data from Figure 2A of luminescence emitted by TETluc transformants SG253, SG255 and wild type grown with 20 μg/ml Dox at different times.

| **Time (h)** | **Strains** |  | **Strains** |  |
| --- | --- | --- | --- | --- |
|  |  | **Wild type** | **SG253** | **SG255** |
| **5** | **Wild type** | — | 0.1093 | 0.3579 |
|  | **SG253** | 0.1093 | — | 0.2886 |
|  | **SG255** | 0.3579 | 0.2886 | — |
| **8** | **Wild type** | — | 0.446 | 0.5029 |
|  | **SG253** | 0.446 | — | 0.9891 |
|  | **SG255** | 0.5029 | 0.9891 | — |
| **21** | **Wild type** | — | 0.4051 | 0.1614 |
|  | **SG253** | 0.4051 | — | 0.5221 |
|  | **SG255** | 0.1614 | 0.5221 | — |
| **25** | **Wild type** | — | 0.3664 | 0.1763 |
|  | **SG253** | 0.3664 | — | 0.6649 |
|  | **SG255** | 0.1763 | 0.6649 | — |
| **28** | **Wild type** | — | 0.4164 | 0.0481 |
|  | **SG253** | 0.4164 | — | 0.166 |
|  | **SG255** | 0.0481 | 0.166 | — |
| **31** | **Wild type** | — | 0.3286 | 0.0795 |
|  | **SG253** | 0.3286 | — | 0.2156 |
|  | **SG255** | 0.0795 | 0.2156 | — |
| **46** | **Wild type** | — | 0.3878 | 0.0795 |
|  | **SG253** | 0.3878 | - | 0.1492 |
|  | **SG255** | 0.0795 | 0.1492 | - |
| **50** | **Wild type** | — | 0.3265 | 0.0092 |
|  | **SG253** | 0.3265 | — | 0.0474 |
|  | **SG255** | 0.0092 | 0.0474 | — |
| **55** | **Wild type** | — | 0.3458 | 0.041 |
|  | **SG253** | 0.3458 | — | 0.1253 |
|  | **SG255** | 0.041 | 0.1253 | — |
| **60** | **Wild type** | — | 0.3796 | 0.0239 |
|  | **SG253** | 0.3796 | — | 0.0763 |
|  | **SG255** | 0.0239 | 0.0763 | — |

**Table S4.** Statistical significance (p-values) obtained after pairwise comparisons of data of luminescence emitted by TETluc transformant SG255 grown at different Dox concentrations from Figure 2B.

| **Time**  **(h)** | **Dox**  **(μg/ml)** | **Dox (μg/ml)** | | | | |
| --- | --- | --- | --- | --- | --- | --- |
|  |  | **0** | **2.5** | **5** | **10** | **20** |
| **5** | **20** | 0.0169 | 0.0430 | 0.0428 | 0.2264 | — |
| **21** | **20** | 0.0001 | 0.0079 | 0.0116 | 0.0281 | — |
| **25** | **20** | 0.0015 | 0.0336 | 0.0293 | 0.0621 | — |
| **30** | **20** | 0.0026 | 0.3984 | 0.1198 | 0.1401 | — |
| **46** | **20** | 0.0015 | 0.5643 | 0.0700 | 0.3913 | — |
| **50** | **20** | 0.0002 | 0.6966 | 0.0118 | 0.0188 | — |
| **55** | **20** | 0.0002 | 0.2614 | 0.0453 | 0.2549 | — |

**Table S5.** Statistical significance (p-values) from comparison of growth SG255 at different times with different Dox concentrations (Figure 2C).

| **Time**  **(h)** | ***p*-value** |
| --- | --- |
| **5** | 0.5505 |
| **21** | 0.5639 |
| **25** | 0.3221 |
| **30** | 0.2902 |
| **46** | 0.3740 |
| **50** | 0.3306 |
| **55** | 0.3109 |

**Table S6.** Statistical significance obtained after pairwise comparisons of carS mRNA data from Figure 3B.

|  |  | | **Control** | | |  | | **Dox** | | |  |
| --- | --- | --- | --- | --- | --- | --- | --- | --- | --- | --- | --- |
|  |  | | **Wild type** | | **SG262** | | | **SG39** | | **SG262** | |
| **Control** | **Wild type** | | — | | 0.359 | | | 0.0296 | | 0.1964 | |
|  | **SG260** | | 0.359 | | — | | | 0.1247 | | 0.1801 | |
| **Dox** | **Wild type** | | 0.0296 | | 0.1247 | | | — | | 0.0809 | |
|  | **SG260** | | 0.1964 | | 0.1801 | | | 0.0809 | | — | |
|  |  | **SG39** | | **SG262** | | | **SG39** | | **SG262** | | |
| **Control** | **SG39** | — | | 0.0461 | | | 0.6641 | | 0.2393 | | |
|  | **SG262** | 0.0461 | | — | | | 0.0071 | | 0.3595 | | |
| **Dox** | **SG39** | 0.6641 | | 0.0071 | | | — | | 0.129 | | |
|  | **SG262** | 0.2393 | | 0.3595 | | | 0.129 | | — | | |

**Table S7.** Statistical significance (p-values) obtained for carRA mRNA in SG39 and SG262 with 20 Dox. (Data from Figure 4A).

|  |  | **Control** | |  | **Dox** | |  |
| --- | --- | --- | --- | --- | --- | --- | --- |
|  |  | **SG39** | **SG262** | | **SG39** | **SG262** | |
| **Control** | **SG39** | — | 0.199 | | 0.3163 | 0.0708 | |
|  | **SG262** | 0.199 | — | | 0.193 | 0.4061 | |
| **Dox** | **SG39** | 0.3163 | 0.193 | | — | 0.0426 | |
|  | **SG262** | 0.0708 | 0.4061 | | 0.0426 | — | |

**Table S8.** Statistical significance (p-values) obtained for carB mRNA in SG39 and SG262 with 20 Dox.

(Data from Figure 4A).

|  |  | **Control** | |  | **Dox** | |  |
| --- | --- | --- | --- | --- | --- | --- | --- |
|  |  | **SG39** | **SG262** | | **SG39** | **SG262** | |
| **Control** | **SG39** | — | 0.4687 | | 0.8164 | 0.0714 | |
|  | **SG262** | 0.4687 | — | | 0.5606 | 0.2835 | |
| **Dox** | **SG39** | 0.8164 | 0.5606 | | — | 0.0485 | |
|  | **SG262** | 0.0714 | 0.2835 | | 0.0485 | — | |

**Table S9.** Statistical significance (p-values) for comparison of carotenoid data from SG39 and SG262 grown with 20 Dox. (Data from Figure 4B).

|  | |  | **Control** | | **Dox** | |  |
| --- | --- | --- | --- | --- | --- | --- | --- |
|  |  | | **SG39** | **SG262** | **SG39** | **SG262** | |
| **Control** | **SG39** | | — | 0.0938 | 0.189 | 0.0175 | |
|  | **SG262** | | 0.0938 | — | 0.0147 | 0.0072 | |
| **Dox** | **SG39** | | 0.189 | 0.0147 | — | 0.0001 | |
|  | **SG262** | | 0.0175 | 0.0072 | 0.0001 | — | |

**Table S10.** Statistical significance (p-values) from comparison of RT-PCR data of carS expression from SG39 and SG262 grown with 10 Dox. (Data from Figure 6A).

|  |  |  | **Control** |  |  | **Dox** | |  |
| --- | --- | --- | --- | --- | --- | --- | --- | --- |
|  |  | **Wild type** | **SG39** | **SG262** | **Wild type** | **SG39** | | **SG262** |
| **Control** | **Wild type** | — | 0.1942 | 0.1156 | 0.4343 | | 0.1212 | 0.0474 |
|  | **SG239** | 0.1942 | — | 0.1372 | 0.2995 | | 0.9452 | 0.0126 |
|  | **SG262** | 0.1156 | 0.1372 | — | 0.0724 | | 0.1351 | 0.0511 |
| **Dox** | **Wild type** | — | 0.2995 | 0.0724 | — | | 0.2026 | 0.0118 |
|  | **SG39** | 0.1212 | — | 0.1212 | 0.2026 | | — | 0.0126 |
|  | **SG262** | 0.0474 | 0.0126 | — | 0.0118 | | 0.0126 | — |

**Table S11.** Statistical significance (p-values) from comparison of RT-PCR data of mluc expression from SG253 and SG255 grown with 10 Dox. (Data from Figure 6B).

|  |  | **Control** | | **Dox** | |
| --- | --- | --- | --- | --- | --- |
|  |  | **SG253** | **SG255** | **SG253** | **SG255** |
| **Control** | **SG253** | — | 0.2403 | 0.1308 | 0.3999 |
|  | **SG255** | 0.2403 | — | 0.2961 | 0.4097 |
| **Dox** | **SG253** | 0.1308 | 0.2961 | — | 0.356 |
|  | **SG255** | 0.3999 | 0.4097 | 0.356 | — |

**Table S12.** Statistical significance (p-values) from comparison of RT-PCR data of carRA expression from SG39 and SG262 grown with 10 Dox. (Data from Figure 6C).

|  |  |  | **Control** |  |  | **Dox** | |  |
| --- | --- | --- | --- | --- | --- | --- | --- | --- |
|  |  | **Wild type** | **SG39** | **SG262** | **Wild type** | **SG39** | | **SG262** |
| **Control** | **Wild type** | — | 0.0954 | 0.0214 | 0.5131 | | 0.0330 | 0.0992 |
|  | **SG39** | 0.0954 | — | 0.0505 | 0.0234 | | 0.7338 | 0.0234 |
|  | **SG262** | 0.0138 | 0.0505 | — | 0.0012 | | 0.0065 | 0.0142 |
| **Dox** | **Wild type** | — | 0.0234 | 0.0012 | — | | 0.0027 | 0.0344 |
|  | **SG39** | 0.033 | — | 0.0065 | 0.0027 | | — | 0.0029 |
|  | **SG262** | 0.0992 | 0.0234 | — | 0.0344 | | 0.0029 | — |

**Table S13.** Statistical significance (p-values) from comparison of RT-PCR data of carB expression from SG39 and SG262 grown with 10 Dox. (Data from Figure 6D).

|  |  |  | **Control** |  |  | **Dox** | |  |
| --- | --- | --- | --- | --- | --- | --- | --- | --- |
|  |  | **Wild type** | **SG39** | **SG262** | **Wild type** | **SG39** | | **SG262** |
| **Control** | **Wild type** | — | 0.1233 | 0.0138 | 0.1798 | | 0.0720 | 0.0045 |
|  | **SG39** | 0.1233 | — | 0.1615 | 0.0365 | | 0.7338 | 0.0379 |
|  | **SG262** | 0.0138 | 0.1615 | — | 0.0005 | | 0.0484 | 0.0142 |
| **Dox** | **Wild type** | — | 0.0365 | 0.0005 | — | | 0.0127 | 0.0014 |
|  | **SG39** | 0.072 | — | 0.0484 | 0.0127 | | — | 0.0131 |
|  | **SG262** | 0.0045 | 0.0379 | — | 0.0014 | | 0.0131 | — |

**Table S14.** Statistical significance (p-values) from comparison of RT-PCR data of carS expression from wild type, OEcarS transformants SG263 and SG264. (Data from Figure 7C).

|  |  |  | **Dark** |  |  | | **Light** |  |
| --- | --- | --- | --- | --- | --- | --- | --- | --- |
|  |  | **Wild type** | **SG263** | **SG264** | **Wild type** | **SG263** | | **SG264** |
| **Dark** | **Wild type** | — | 0.0026 | 0.0332 | 0.0404 | 0.0140 | | 0.0316 |
|  | **SG263** | 0.0026 | — | 0.2244 | 0.0163 | 0.0485 | | — |
|  | **SG264** | 0.0332 | 0.2244 | — | 0.0432 | 0.3939 | | 0.0501 |
| **Light** | **Wild type** | — | 0.0163 | 0.0432 | — | 0.009 | | 0.0235 |
|  | **SG263** | 0.014 | — | 0.3939 | 0.009 | — | | 0.7944 |
|  | **SG264** | 0.0316 | — | — | 0.0235 | 0.7944 | | — |

**Table S15.** Statistical significance (p-values) from comparison of RT-PCR data of carRA expression from wild type, OEcarS transformants SG263 and SG264. (Data from Figure 7D).

|  |  |  | **Dark** |  |  | | **Light** |  |
| --- | --- | --- | --- | --- | --- | --- | --- | --- |
|  |  | **Wild type** | **SG263** | **SG264** | **Wild type** | **SG263** | | **SG264** |
| **Dark** | **Wild type** | — | 0.0008 | 0.0012 | 0.0377 | 0.4146 | | 0.9566 |
|  | **SG263** | 0.0008 | — | 0.6095 | 0.0074 | 0.0759 | | 0.0299 |
|  | **SG264** | 0.0012 | 0.6095 | — | 0.0074 | 0.0299 | | 0.3145 |
| **Light** | **Wild type** | — | 0.0074 | 0.0074 | — | 0.0075 | | 0.0075 |
|  | **SG263** | 0.4146 | — | 0.0299 | 0.0075 | — | | 0.6884 |
|  | **SG264** | 0.9566 | 0.2584 | — | 0.0075 | 0.6884 | | — |

**Table S16.** Statistical significance (p-values) from comparison of RT-PCR data of carB expression from wild type, OEcarS transformants SG263 and SG264. (Data from Figure 7E).

|  |  |  | **Dark** |  |  | **Light** |  |
| --- | --- | --- | --- | --- | --- | --- | --- |
|  |  | **Wild type** | **SG263** | **SG264** | **Wild type** | **SG263** | **SG264** |
| **Dark** | **Wild type** | — | 0.6406 | 0.9755 | 0.0121 | 0.2385 | 0.2263 |
|  | **SG263** | 0.6406 | — | 0.6353 | 0.0025 | 0.0651 | 0.4557 |
|  | **SG264** | 0.9755 | 0.6353 | — | 0.0024 | 0.0299 | 0.1719 |
|  | **Wild type** | — | 0.0025 | 0.0024 | — | 0.0027 | 0.0026 |
| **Light** | **SG263** | 0.2385 | — | 0.0299 | 0.0027 | — | 0.7724 |
|  | **SG264** | 0.2263 | 0.4557 | — | 0.0026 | 0.7724 | — |

**Table S17.** Statistical significance (p-values) from comparison of carotenoid accumulation from wild type, OEcarS transformants SG263 and SG264. (Data from Figure 7B).

|  | |  |  | **Dark** |  |  | | **Light** |  |
| --- | --- | --- | --- | --- | --- | --- | --- | --- | --- |
|  |  | | **Wild type** | **SG263** | **SG264** | **Wild type** | | **SG263** | **SG264** |
| **Dark** | **Wild type** | | — | 0.0085 | 0.0087 | 0.009 | 0.0081 | | 0.0087 |
|  | **SG263** | | 0.0085 | — | 0.8403 | 0.0002 | 0.4248 | | 0.7119 |
|  | **SG264** | | 0.0087 | 0.8403 | — | 0.0002 | 0.6234 | | 0.7874 |
| **Light** | **Wild type** | | — | 0.0002 | 0.0002 | — | 0.0002 | | 0.0002 |
|  | **SG263** | | 0.0081 | — | 0.6234 | 0.0002 | — | | 0.1992 |
|  | **SG264** | | 0.0087 | 0.7119 | — | 0.0002 | 0.1992 | | — |
